# Equality, Efficiency, and Developmental Education Reform: The Impact of SB 1720 on the Mission of the Florida College System

**DOI:** 10.1177/0091552119876327

**Published:** 2019-09-27

**Authors:** Amanda N. Nix, Tamara Bertrand Jones, Rebecca L. Brower, Shouping Hu

**Affiliations:** 1 Florida State University, Tallahassee, USA

**Keywords:** community colleges, developmental education, institutional mission, qualitative research

## Abstract

**Objective:**

Community colleges have long made higher education more accessible to students from diverse academic backgrounds, particularly those who are academically underprepared and require remediation. In light of developmental education (DE) reform, our article answers the following questions: How do campus personnel articulate the unique mission of Florida’s state colleges, formerly known as community colleges? Furthermore, how do they perceive the mandates of reform to have shaped their ability to carry out this mission?

**Method:**

This work is based on an embedded case study of 10 Florida College System institutions. Qualitative data were gathered between 2014 and 2018 from 544 college presidents, administrators, faculty, staff through 92 focus groups and 8 interviews.

**Results:**

Campus personnel strongly affirmed the mission of the Florida Colleges System as one of democratic equality. However, many were concerned that DE reform, namely Senate Bill 1720, prioritized efficiency over equality in the pursuit of cost savings. Specifically, participants expressed frustration that reforms accelerated DE coursework to an unmanageable pace and ignored the presence of a digital divide. Opinions of DE reform improved in the 4 years following implementation, but some concern persisted.

**Contributions:**

Our findings highlight the centrality of democratic equality to the community college mission for campus personnel. They also suggest that equality and efficiency need not always be opposing goals in education reform. Finally, they call into question social policy that universally promotes accelerated and computer-based courses without considering that some students may require accommodations.

Developmental education (DE), also known as postsecondary remediation, has been the focus of public criticism in recent years. Those most vocal against it are primarily concerned with the high costs of DE and low number of students successfully moving through such programs into college-level coursework (H. M. Levin & Calcagno, 2008; Smith, 2015). Of the two thirds of college students who are referred to DE courses, less than one half complete them within 3 years of initial enrollment (Bailey, Jeong, & Cho, 2010). In response, educational reform groups have advocated for a number of revisions to DE, including more accurate measures of assessment for course placement, acceleration, and improved curriculum and pedagogy (Fain, 2012; Jaggars & Bickerstaff, 2018).

In 2013, the Florida legislature passed their own version of reform—Senate Bill 1720 (SB 1720)—in an effort to address both the concerns of cost and completion rates. Under the legislation, placement tests were made optional for those who graduated with a standard diploma from a public Florida high school in 2007 or later and for active-duty service members. In turn, these students were given the option to bypass DE and enroll directly in college-level English and mathematics courses. The legislation also required colleges to increase advising services, enhance academic supports, and redesign developmental-level course pedagogy so that students could choose between different delivery formats (compressed, corequisite, contextualized, and modularized).

SB 1720 generated significant change within the Florida College System (FCS)^1^ over a very short period of time. It is within this historic context that we pose the following research questions:

**Research Question 1:** How do campus personnel articulate the unique mission of Florida’s state colleges (formerly known as community colleges)?

**Research Question 2:** How do campus personnel perceive the mandates of SB 1720 to have shaped their ability to carry out this mission at their respective institutions in the 4 years following its passage?

In this article, we present how presidents, faculty, advisors, administrators, and other campus personnel thought SB 1720’s focus on social efficiency limited their ability to successfully fulfill a mission of promoting educational access and success for all. We also discuss how these perspectives have changed over time and whether equality and efficiency might be complementary outcomes of SB 1720, rather than opposing ones.

## Theoretical Framework

The theoretical framework for this article is based on the work by David Labaree (1997) regarding the competing goals of education. When scholars speak about the ‘mission’ of the community college, they are often referring to the numerous curricular functions these institutions fulfill, including academic transfer preparation, vocational education, DE, contract or continuing education, and community service (Bailey & Averianova, 1998; Cohen, Brawer, & Kisker, 2014). At different points in time, the emphasis placed on each function has varied (Bragg, 2001; Brint & Karabel, 1989; J. S. Levin, 2000). Today, community colleges are comprehensive, often attempting to serve some combination of all 5 competing functions simultaneously (Bailey & Averianova, 1998; Barringer & Jaquette, 2018; Cohen et al., 2014). Illustrating this point about the comprehensive nature of community colleges, the current mission statement of the FCS (n.d.) is as follows: ‘to provide access to high-quality, affordable academic and career educational programs that maximize student learning and success, develop a globally competitive workforce and respond rapidly to diverse state and community needs’ (para. 2).

Amid these various functions, David Labaree (1997) identifies 3 competing goals of education: democratic equality, social mobility, and social efficiency. The first goal, democratic equality, emphasizes that schools exist to create citizens and facilitate relative equality. This is accomplished via citizenship training, equal treatment of students from diverse backgrounds, and equal access to education at any level. Proponents of DE curriculum consider remediation an integral part of providing equal access to post-secondary education and credentials for students who arrive to college unprepared for college-level work (Bahr, 2010). Advocates of social mobility, the second goal, view education and, more specifically, credentials as a commodity that can be used to help individuals compete in the workplace. Although the goals of democratic equality and social mobility are often at odds, there is one thing upon which these perspectives can agree—that equal access to education is important. As such, Labaree (1997) notes that proponents of democratic equality and social mobility often come together to form a political coalition that advances a ‘progressive’ educational agenda.

The third goal, social efficiency, focuses on the connection between schools and economic growth. Social efficiency is commonly concerned with using public funds wisely. Labaree’s (1997) conception of social efficiency is revealed in the streamlining and scaling back of DE in an effort to reduce time to degree and the overall cost of education to students and taxpayers. Those who value social efficiency, often policy-makers, employers, and college administrators, comprise a second coalition committed to market-oriented ideals that stand in contrast to the aforementioned progressive agenda.

Two of these goals—democratic equality and social efficiency—are generally categorized as public goods, whereas the third (social mobility) is considered a private good (Labaree, 1997). Although community colleges play an important role in advancing both public and private goods, this article focuses specifically on the tension present between the public goods of democratic equality and social efficiency. Additional rationale for highlighting just 2 goals, rather than all 3, comes from their relative importance to participants in the study. The data we present demonstrate how campus personnel were primarily concerned with the interaction between social efficiency and the availability of educational opportunities for historically underserved students, much more than social mobility. In the literature review that follows, we first discuss the goals of community college education in terms of democratic equality and social efficiency and then we move to an examination of contemporary reform efforts.

## Literature Review

### The Mission of Community Colleges

Traditionally, the mission of community colleges has been one of democratic equality (Brint & Karabel, 1989; Dowd, 2003; Labaree, 1997). Community colleges have long been known for making higher education more available to all, especially nontraditional, low-income, female, and underrepresented racial or ethnic minority students (National Center for Education Statistics, 2008). Within the FCS (n.d.), we see references to these aspects of equality in the mission statement cited above: ‘to provide *access* to high-quality, affordable academic and career educational programs [emphasis added]’ (para. 2).

Community colleges facilitate access through open admissions policies and relatively low tuition and fees (Phillippe & Patton, 2000; Schudde & Goldrik-Rab, 2015). Open admission policies allow students to complete an informal admissions process that only requires them to provide documentation of a GED (General Educational Development) or high school diploma (National Center for Education Statistics, 2008). Other college admissions tests, such as the SAT (Scholastic Aptitude Test) and ACT (American College Testing), are considered optional. Regarding affordability, in Florida, it is twice as expensive to attend a public state university and 5 to 6 times more expensive to attend a private college than an FCS institution (Division of Florida Colleges, 2014). These tuition differences are stark, highlighting the important role that FCS institutions play in advancing democratic equality for low-income students. Once students are enrolled, community colleges continue to ‘provide access to high-quality, affordable academic and career educational programs’ (FCS, n.d., para. 2) through DE curriculum and academic support services. These secondary components of access are intended to help underprepared students make the most of their ability to enroll in college by supporting the ongoing pursuit of postsecondary credentials.

As community colleges begin to take a more market-oriented approach to education, there is now increased focus on economic principles such as competition, productivity, and efficiency (Dowd, 2003; Giroux, 2002). Institutional policies and procedures, research, and state and national legislative agendas have responded accordingly (Giroux, 2002; Slaughter & Rhoades, 2009). In a large-scale, qualitative study much like ours, J. S. Levin (2001) noted that contemporary, higher education reforms focus almost exclusively on economic development, competition, and efficiency.

### DE

One aspect of education that has seen significant reform in recent years is postsecondary remediation. DE, as a formal program, was first offered in 1849 at the University of Wisconsin. Since that time, a number of national events and policies have influenced the composition of DE enrollees including World War II, the passage of the Civil Rights Act of 1964, the Higher Education Act of 1965, and Title IX. Collectively, these historic moments increased the number of veterans, females, and racial and ethnic minorities choosing to enroll in college between the 1940s and 1980s (see Merisotis & Phipps, 2000, for a complete history). In summary, ‘remediation in colleges and universities is not an appendage with little connection to the mission of the institution but represents a core function of the higher education community that it has performed for hundreds of years’ (Merisotis & Phipps, 2000, p. 79).

National debate about the role of DE ensues. Critics often cite cost as a major concern, arguing that DE bills taxpayers twice for the teaching of high school–level math and English course content (Boylan, 1999; Merisotis & Phipps, 2000; Saxon & Boylan, 2001). Because students did not learn the material in high school, taxpayers provide them a second chance in the form of DE. In Florida, the location of this study, as many as 70% of first-time-in-college (FTIC) students needed developmental coursework in at least one subject area during the 2009-2010 academic year. Those remediation efforts cost the state US$81 million in taxpayer dollars (Underhill, 2013). In response, proponents of DE counter that remedial curriculum is inexpensive relative to the number of students who benefit; in 2004-2005, the national cost of DE was approximately 0.48% of the public higher education budget, or US$1.13 billion of US$234.8 billion (Pretlow & Wathington, 2012).

In addition to cost, there is much debate about the extent to which DE actually facilitates democratic equality. Critics highlight the fact that Black and Hispanic students are disproportionately enrolled in DE coursework (Attewell, Lavin, Domina, & Levey, 2006; Complete College America, 2012) and fail to complete the work at higher rates than their White peers (Bahr, 2010; Bailey et al., 2010). Some also question the validity of high-stakes placement tests and the arbitrary cut scores used to make DE course enrollment decisions (Scott-Clayton, 2012; Scott-Clayton, Crosta, & Belfield, 2014). Advocates of DE disagree, saying that these programs facilitate democratic equality by allowing more diverse groups of students to pursue higher education than could otherwise (Bahr, 2010). They fear that proposals to remove or limit DE may negatively affect the educational attainment of already underserved students (Bahr, 2004; Lavin & Weininger, 1998).

### SB 1720

The provisions of SB 1720 address the inefficiencies and inequities of traditional DE models. For instance, when explaining the low completion rates of DE, scholars sometimes point to a pipeline effect, whereby students leak out of lengthy remedial pathways between classes, often before getting to college-level work (Charles A. Dana Center, 2016; Hern, 2010). In response, national reform efforts commonly advocate for accelerating curriculum in an effort to achieve efficiency (Jaggars & Bickerstaff, 2018). SB 1720 is no exception. Instead of traditional, 16-week remedial courses, FCS institutions were required by the bill to offer students a choice of DE courses taught in some of the following 4 modalities: compressed, contextualized, corequisite, and modularized. Two in particular—compressed and modularized courses—place a heavy emphasis on acceleration.

In 2015, DE courses in Florida were most commonly taught in a compressed environment (Hu et al., 2015). This modality involves condensing a DE course into a shorter time frame (e.g., 4-, 8-, or 12-week classes) or combining 2 classes into one, 16-week semester (e.g., MAT 0018 and 0028). Modularized classes, the second most common modality (Hu et al., 2015), utilize diagnostic tests and units so that students can focus specifically on their areas of weakness. Early exit options sometimes exist for students who complete the necessary coursework faster than their peers.

Studies of the Accelerated Learning Program, California Acceleration Project, and other programs lend support to the practice of accelerating remediation. DE taught as compressed and corequisite courses are particularly helpful in moving underprepared students into college-level courses (Hern, 2010; Jaggars, Hodara, Cho, & Xu, 2015). The effects of modularized courses are positive as well, yet modest (Bickerstaff, Fay, & Trimble, 2016).

SB 1720 also emphasized the incorporation of technology into classroom instruction and out-of-class support services. As DE enrollments were expected to decrease during implementation, web-based tutoring and writing help, virtual advising and orientation sessions, and instructional software were offered as a way to address issues that may emerge. Research on some of these specific programs is limited, but e-learning, more broadly, is found to be as effective as traditional learning, and in some cases more so (Bell & Federman, 2013). However, Bell and Federman (2013) point out that unequal access to and usage of technology has been shown to present a barrier for low-income and underprepared students in their pursuit of education.

To date, the documented impact of SB 1720 on student outcomes has been fairly positive. Although pass rates in individual gateway courses have decreased at FCS institutions, enrollment in those gateway courses has increased so much so that the total number of incoming students who pass gateway courses has increased (Hu et al., 2016). In addition, some quantitative data show that the bill has made strides toward greater democratic equality. Black students, in particular, have benefited from the ability to enroll directly in college-level courses, narrowing the Black–White achievement gap in college gateway courses (Hu et al., 2016; Park et al., 2018). Data also indicate that the bill has improved social efficiency by saving students money (Division of Florida Colleges, 2015; Mokher, Park, & Hu, 2019). The Division of Florida Colleges (2015) attributes these savings to a reduction in tuition and fees spent on DE course sequences and an increase in academic support services offered at little or no cost to students.

This qualitative analysis takes a different perspective on the tension between equality and efficiency, giving voice to the lived experiences of campus personnel over a 4-year period. We first sought to understand how campus personnel conceptualize the mission of the FCS: as a system of institutions that prioritize equality, efficiency, or both. We were also interested in how such individuals felt SB 1720 shaped their ability to pursue this mission, particularly over time.

## Method

To answer the questions at hand, we utilized qualitative data gathered from individuals employed by 10 FCS institutions between 2014 and 2018 to conduct an embedded, single-case study about the perceived influence of SB 1720 on the mission of Florida’s state colleges.^2^ In line with other embedded case studies, this research focuses on multiple units of analysis, namely, individual-level perspectives of administrators, faculty members, advisors, and college presidents, embedded within one case (the FCS; Yin, 2013). To capture the diversity of opinion across the state, our sample included institutions that varied greatly, in terms of institutional size and complexity, geography, and the demographic make-up of the local community.

### Data Collection

To generate our sample, email invitations were sent in the summer of 2014 to administrators at all 28 FCS institutions requesting their participation in our research. Ten institutions accepted the invitation and subsequently hosted research teams for 2 days in the fall of 2014 and early spring of 2015. Between the fall of 2015 and spring of 2018, we returned to 8 of these colleges to gain a sense of how the perceptions of the legislation held by administrators, faculty members, advisors, and other campus personnel might have changed. We returned to individual colleges between 1 and 3 additional times, depending on our research schedule, available financial resources, and colleges’ willingness to participate in continued data collection efforts. In total, our findings reflect data collected during 23 site visits to the 10 colleges that comprise our sample.^3^

Each institution assisted researchers with the logistics of the initial and follow-up site visits, including soliciting potential focus group participants from the following groups of stakeholders: students, faculty, staff, and reform implementation team members (primarily administrators). At least 2 researchers participated in each of the 23 visits and generated field notes, recording salient, interesting, or illuminating observations for later consideration. In the first year, researchers conducted 54 focus groups with 323 campus personnel at these 10 institutions. To the extent possible, focus group sessions centered around one kind of campus personnel (e.g., advisors or faculty members) and were guided loosely by a list of questions relevant to the population at hand. Some of the questions asked of faculty related to curriculum redesign; for advisors, placement procedures; and for administrators, the campuswide impact of SB 1720 on finances and technology. Follow-up visits between 2015 and 2018 produced an additional 38 focus group sessions and 4 interviews with a total of 217 campus personnel employed by 8 of the 10 original colleges. Phone interviews were also conducted with 4 of the 10 college presidents in 2015 to gather additional perspectives. All focus group sessions and interviews were audio-recorded and then transcribed with consent of the participants.

Although the data we present are drawn exclusively from the semi-structured focus groups and interviews, 2 additional kinds of data—institutional documents provided by the colleges and field observations gathered during site visits—served an important role in shaping our thinking and analysis. For example, in an earlier phase of the project, researchers analyzed Implementation Plans from all 28 FCS institutions to identify similarities, differences, and innovative practices that warranted a deeper look during site visits. These plans were also used to support the development of the focus group interview protocol and the coding framework used for data analysis.

### Data Analysis

In Year 1, data analysis involved a 5-person research team. We began the coding process with 157 *a priori codes* covering a variety of topics discussed by the focus groups, including college access, reported student outcomes, the impact of reform on specific student populations, and perceptions, both positive and negative, of SB 1720. We first coded a subset of the data files for the reliability-building process. Discussions of emerging themes resulted in 54 additional codes. Once the coding structure was solidified, we then worked collaboratively to code the remaining data. Analytic memos were generated to document important findings that required further exploration.

Between 2015 and 2018, we revisited 8 of the institutions to gauge the extent to which the perspectives of campus personnel and students were persistent or variable. The data collected on these follow-up visits were analyzed in the same way as data collected on the initial site visit. The coding structure grew from year to year to incorporate emergent codes but, otherwise, the team-based structure and reliability-building process remained consistent.

During our first round of coding, the team made use of descriptive and process coding (Saldaña, 2009) to analyze transcripts from the 2014-2015 academic school year. Because of the complexity of our data, we also engaged in simultaneous coding, whereby paragraphs of text were assigned a number of relevant codes (Saldaña, 2009). This was a particularly useful tactic, considering our broad research questions and the interrelationship between many of our codes.

Through a second round of coding, we were able to reorganize our data from academic year 2014-2015 to tell a cohesive story about campus personnel’s commitment to the mission of democratic equality. We then made use of longitudinal coding, as described by Saldaña (2009), to determine which of the themes increased, deceased, or remained consistent in data collected between 2015 and 2018. In addition, researchers searched for examples of disconfirming evidence in an effort to reduce research bias.

It is worth noting that the focus group and interview protocols were originally designed to gauge individual sentiments about the legislation’s passage and identify innovative advising and instructional practices that arose from implementation of the bill. Participants were not asked questions directly related to institutional mission on the first site visit. The impetus for this article arose out of countless, impromptu mentions of college mission while campus personnel talked about the impacts of the bill, both positive and negative, on their daily work. The quotations presented below are expository exemplars intended to shed light on these findings.

Data and analyst triangulation were important for establishing the trustworthiness of our findings. To minimize individual researcher subjectivity, we also employed systematic, iterative coding approaches to establish trustworthiness (Strauss & Corbin, 1990). At the conclusion of data analysis, member checks were conducted with participants to confirm the accuracy and clarity of our interpretations. Our final step involved debriefing with peers; we consulted with colleagues who were intimately familiar with DE reform and encouraged them to question our interpretations as *devil’s advocates*, or *critical friends* (Miles, Huberman, & Saldaña, 2014; Patton, 2015).

## Findings

### Perceptions of Mission

Throughout all 4 years of data collection, campus personnel clearly articulated the unique mission of community colleges as institutions dedicated to democratic equality. Campus personnel spoke about this topic of ‘mission’ in 24 of the 100 transcripts, often without being explicitly asked about it. We heard from presidents, faculty, and staff alike that FCS institutions fill a critical role in serving a diverse group of students, especially those who have no other options for postsecondary education. To this point, one faculty member described the student population at his college as being different than the student population at more selective, 4-year institutions, particularly in terms of academic preparation: ‘We are not getting the A and B students. They are going elsewhere. We are getting those ‘barely made a C,’ or the D students.’ Despite the inherent challenges, he confirmed an institutional commitment to the academic success of such students: ‘Our mission says, ‘We welcome you. Wherever you are on the continuum, we will take you where you are, and we will help you.’’

Previously, developmental-level coursework helped students at varying points on the continuum become college-ready. The initial consensus of campus personnel was that some of the mandates in SB 1720, including the reduction and acceleration of DE, were ‘counter’ or ‘disruptive’ to a democratic mission. A faculty member shared, with conviction:

We exist to help those students who didn’t have the skill level to get into a university, who didn’t have the money, who needed more time. We’re going against everything that we were created to do [by implementing SB 1720]. And then we’re getting presentations made to us by [education reform] groups … They’re trying to get us to do with our students what they do with university students. We’re not a university. That’s not what we do … Does the legislature understand why we [community colleges] were created in the first place in 1901? Why we exist?

Immediately following the passage of SB 1720, campus personnel felt that statewide efforts to scale back DE and accelerate coursework were misplaced. They argued that students enrolled at FCS institutions often need more support and more time to get up to speed academically, rather than less. Now, one administrator explained, because of SB 1720, students who arrive to her college in need of remediation no longer receive it. Instead, these students find themselves in college-level courses that are much too difficult:

We see students that come to us severely underprepared. So, to me, it [SB 1720] was like … completely under-cutting the mission of the community college. We are here to prepare students, and they are going to be sitting in their college English class, their first semester, and they are not going to be ready for it.

This new teaching environment proved frustrating for many campus personnel because they felt it did not reflect their own values and goals for education. As one administrator aptly stated, ‘This is not why I came to community college education. If I wanted to be like this, I would have gone to private, 4-year education, or something. This is not our mission.’

In their critiques of SB 1720, campus personnel elaborated on how some of the mandates of SB 1720, namely acceleration and an emphasis on technology, reflected a clear shift toward social efficiency. By decreasing the number of semesters students spent in DE, reformers hoped that individuals and colleges alike would make better use of limited resources such as time and money. One administrator acknowledged this intent of SB 1720, but also pointed out some of the resulting challenges:

I looked at it [SB 1720] from an economic perspective thinking, ‘Okay, here is how they are going to try to save money’ … This was their magic pill to get everybody in and out in 2 years … And, I felt like they just disregarded what the student needed, and what the instructor was able to do. Maybe there was a small portion of me, as a Florida taxpayer, that thought, ‘Okay, good. This will save money in the long run.’ But, the bigger part of me, the educator, said, ‘Oh, no. This is doomed to fail.’

A faculty member at another college echoed these sentiments about the financial motives of SB 1720, saying the bill was ‘a financial decision to save the state tons of money.’ He continued, explaining that this focus on cost savings has been ‘changing a lot of people’s lives, … causing some people … to leave college when they might not have left had they entered it correctly.’ Considering the well-documented literature about low levels of academic success within DE curriculum, this perspective cannot be taken entirely at face value. Even so, this participant’s concern about the prioritization of efficiency over democratic equality is duly noted.

Other participants highlighted the value of helping just a few community college students, per their mission, even when it was not the most efficient course of action. One faculty member articulated this perspective, saying,

We want to give them [students] an opportunity … Out of a hundred people, maybe 10-20 will actually take advantage of it [DE]. But that’s 10-20 more than there would have been before … The legislature sees that 90 or 80 that don’t make it. But what about the 10-20 that do make it? That’s more than it would have been before. And I think that’s what we—our purpose was always for.

Return visits to 8 of the original 10 colleges revealed some changes in perception over time. During the first year of data collection, participants expressed almost universal feelings of shock, anger, dismay, confusion, and panic related to the passage of the bill. Others said they ‘expected the worst.’ In many instances, these worst-case scenarios never came to fruition. A number of participants reflected later that ‘it’s not as bad as I thought.’

During the third and fourth years of data collection, we encountered a number of participants with improved opinions of SB 1720 and its effect on student success. One faculty member noted, ‘My first reaction was, this is going to be an unmitigated disaster. My attitude has changed. It’s definitely been tempered.’ Reflecting on student outcomes, he elaborated,

Even though teaching an intermediate Algebra is, at times, very challenging, dealing with those students who come in who don’t have … much mathematical skills to speak of, overall, we are getting students through and our success in our subsequent courses in college algebra has stayed steady.

As exemplified by this quotation, campus personnel at some colleges reported that rates of student success had either continued at or returned to their pre-SB 1720 levels because of the hard work and dedication of instructors and advisors.

An administrator at another college reported that the bill did more than just maintain former levels of student success. According to the data available to him, SB 1720 generated improved outcomes for Hispanic males on campus: ‘We’ve had some really significant changes on a very small number of students, Hispanic males particularly, which has more than doubled their success rate.’ Comparable gains were not mentioned by those at other institutions but may be present. These qualitative findings broach the possibility that SB 1720 may have found a way to turn democratic equality and social efficiency into complementary, rather than opposing, goals. As one administrator humorously noted,

I felt like Chicken Little, ‘the sky is going to fall… ’ and then it really didn’t… There were a lot of adjustments that had to be made, and I think our college reacted appropriately and well. Turning to the topic of social efficiency, she added,

It hasn’t gotten worse, but I don’t see that it’s much better … As a state, have we saved a lot of money? Because I believe that was part of the impetus to being able to hurry up the time to degree and not make any students take all these classes and getting through the system faster … I don’t have that information … As a taxpayer, rather than an educator, I would say if it’s not gotten any worse and we save $5 million, then it worked.

Although this administrator did not have access to information about state finances, she was willing to consider that the bill was successful, maintaining the former level of democratic equality within the FCS while also improving social efficiency.

For others, however, concerns have either remained consistent or worsened over the 4 years of observation. The campus personnel who were alarmed by the impact of SB 1720 on college mission were concerned that the bill (a) accelerated DE course-work to an unmanageable pace and (b) ignored the digital divide when increasing the use of technology in and outside of the classroom. Participants expressed worry that these components of SB 1720 hindered the pursuit of academic success for some groups of students which, in turn, made it difficult for campus personnel to live out the mission to which they were committed.

### Acceleration: ‘It’s Just Going Too Fast’

DE course sequences are considered by many to be long and drawn out. In some cases, students must complete 3 levels of mathematics remediation before ever enrolling in credit-bearing, college-level work (Boatman & Long, 2018). It is no wonder that many DE students fail to complete these lengthy course sequences and leave college altogether (Bailey et al., 2010; Complete College America, 2012). Accelerating DE curriculum via SB 1720 was an attempt to achieve greater efficiency in the remediation process.

Despite the positive trends related to acceleration documented in the literature, faculty in these data were not shy in sharing their initial disdain of redesigned courses, particularly for those that were compressed. One faculty member, summarizing the sentiments of many others, strongly proclaimed, ‘Do I think that 8 weeks is adequate time to teach Developmental I or II? Absolutely not.’ Another faculty member shared a moving account of watching underprepared students become frustrated with compressed course material:

Right outside my office is a lobby area where students sit and study and meet with the tutor … I see the real frustration that they are feeling as being a part of these compressed classes. The very population that needs additional time, it’s been cut in half. When I first heard about it, it was mind boggling to me as a professor … I understand, on one level, that they thought it might be helpful to help the students get through 2 prep courses or Dev Ed courses in 1 semester. Good idea on the surface. But, when you start digging underneath and seeing the real frustration and seeing students not be successful … you start to think about it.

Much of the concern we documented was rooted in a fear that students are not able to keep pace in redesigned, developmental-level courses. The overwhelming opinion of campus personnel was that these courses simply move too quickly through developmental coursework for students to gain a firm understanding of the material. A faculty member explained the challenge this way:

I find it strange that we are accelerating remediation. To me, you shouldn’t use those 2 words in the same sentence because … when you have a student … [who] really did not do well in high school, and now we’re trying to teach him the same thing that they should have gotten in high school, and compress it, I don’t think it works for those students.

Campus personnel worried that underprepared students need more, rather than less, time in the classroom to fill the gaps in their learning. With colleges speeding up all of DE, primarily in the form of modularized and compressed courses, campus personnel noted that some students were left behind. One advisor reported seeing students ‘take second and third time attempts in the same classes … The legislature’s intent was to reduce the cost, … but these students need … smaller classes and the slow pace.’

Although acceleration can pose a challenge for all students, particular groups were identified as more vulnerable than others. For example, several individuals, including this advisor, voiced concern for English language learners (ELL): ‘I mean, my goodness, I don’t know how a developmental student or a student for whom English is not the first language can do a course in 4 weeks.’ Campus personnel expressed similar sentiments for students with disabilities: ‘I particularly saw with some of our disability students … They did not like that it was 8 weeks and they really couldn’t handle it that way.’ Those simultaneously juggling work, family, and school commitments also faced unique challenges with the design of accelerated courses. As another advisor pointed out, ‘A student [who] is working a full-time job, 40 hours a week, and they have 2 kids maybe shouldn’t be in an accelerated course, because if they get behind 2 classes, they’re behind a week and a half.’ Although ELL, individuals with disabilities, parents, and employed students were not targeted in the language of the bill, participants believed these groups felt the repercussions of acceleration most acutely.

Herein lies an important connection between efficiency-based reform efforts and the mission of FCS institutions to facilitate equality. Compressed and modularized DE courses are certainly more efficient than traditional DE models, reducing student and taxpayer resources invested in remediation. However, campus personnel raised serious concerns about the impact of this efficiency on student outcomes. Although FCS institutions remained open access in name, just as before, campus personnel felt that certain groups of students had trouble translating access to education into academic success and credential attainment due to the pace of redesigned DE curriculum. Because these courses moved too quickly, some students had to retake their DE courses a second or third time, rendering the redesign less efficient and less equal in the end.

Over time, the view some campus personnel held of accelerated courses improved. Although participants were still concerned with other aspects of SB 1720, some ultimately appreciated acceleration. According to one faculty member’s candid remarks, ‘We weren’t happy in the beginning, and in many ways we’re still not happy. We don’t see the program [SB 1720] working well for the students.’ But, to this reflection she added,

On the other hand, one thing that I was initially very skeptical of … was the idea of the 8-week class … And, I have to say, that [acceleration] has surprised me to no end. And I am very glad that we have that.

An advisor echoed these changing sentiments about acceleration, saying, ‘I’ll be the first to say, … I was opposed to pieces of it [SB 1720]. I’m opposed still, … [but] I love the pieces of it where we accelerate [curriculum].’ In both cases, these individuals had witnessed the positive effects of SB 1720 and acceleration for some students’ efficient progression through coursework, allowing them to accrue college credit and build academic momentum much quicker than in the past.

Having considered how campus personnel perceived acceleration to have affected how they pursue a mission of democratic equality, we now move to the second concern shared by participants: a digital divide.

### Digital Divide: ‘The ‘Haves’ and the ‘Have Not’s’’

Technology is commonly associated with improved efficiency. SB 1720 encouraged increased use of technology by FCS institutions, particularly in the form of modularized classes that rely heavily on software and online student services (e.g., advising, orientation, and tutoring). Campus personnel and students expressed concern that this shift placed certain individuals at a disadvantage, either because they did not have access to technology or were uncomfortable using it.

Regarding access to technology, the data indicated that some students do not have reliable computers, Internet connections, or accessories like printers. This digital divide, while prevalent throughout the state of Florida, was most salient for participants in rural communities. An advisor, working in one such community, vividly described the lengths to which students must go to engage in online learning:

We are a very rural county and most people [have] limited access and spotty access to computers. I mean they’ll sit in McDonald’s or Dunkin Donuts parking lot to try to pick up what they need on Wi-Fi … because they don’t have that kind of connectivity. They still have dial-up, you know, in the double wide.

In addition to rural communities, there were student populations highlighted in the data for whom access to technology presented a challenge: returning adults and low-income students. Speaking about the former, one faculty member stated that the ‘older generation doesn’t do computers. Some of them don’t even own [a] computer, … and they don’t have high speed internet.’ A faculty member from a different college shared a similar sentiment about low-income students:

We have so many first time in college students … Many of them are from low, lower socioeconomic homes. They don’t have computers at home so if you are giving homework that’s on a computer it’s, it’s not helpful for them … It makes it difficult and they also see that the difference that [exists] between the ‘haves’ and the ‘have nots’ when you have so many students that have the computers at home and they don’t.

Computers are available for student use in college labs and libraries across the state, although their number and availability varies. According to one staff member, a learning specialist, her campus had ample technological resources for students: ‘Between the learning lab and the library we probably have 100 computers … so there are plenty. If a student comes in and they can’t find a computer, they are not looking hard enough.’ Making use of such resources requires students to spend time on campus, which is often difficult due to family responsibilities, paid employment, and unreliable transportation. One student also made mention of a laptop lending program at his institution for a select group of students. This kind of program was neither widespread among the FCS nor were other students on the same campus aware of it.

Even when students have access to technology, knowledge about how to utilize this technology for academic pursuits is often missing. Faculty members spoke about computer applications such as email, Blackboard, and One Drive causing students trouble in the classroom. One such faculty member shared her surprise with the deficit in computer literacy for some in this way:

I was kind of surprised at how many times I had to explain like how to attach files to an email, how to send an email… Simple stuff like, let me show you how to get through Blackboard [the college’s learning management system]. I do a quick tutorial, and I will get 20 emails that night they don’t know what to click on.

To address technology-related knowledge gaps, faculty described spending valuable class time to cover skills not related to their content area. Advisors at several colleges labeled this challenge of simultaneously teaching math or English *and* computer skills to underprepared students as a ‘double-edged sword’ and a ‘double whammy.’

The concern expressed by our participants was not simply that some students do not have computers or the skills to use them, as this is nothing new to the community college environment. There are many academically prepared students in 2- and 4-year colleges who face similar constraints. Instead, campus personnel worried that the digital divide ultimately affected the ability of academically underprepared students to succeed and progress through higher education, considering the emphasis of SB 1720 on technology.

There was also some concern over the cost of required, classroom technology for low-income students enrolled in modularized and compressed DE courses. Although traditional, print textbooks can be shared among students, checked out at the library, or purchased used, the same is not true of software like McGraw Hill’s ALEKS program and Pearson’s MyMathLab. Implementation of these instructional tools, as they currently stand, may serve as an impediment in the FCS’s pursuit of democratic equality for low-income students.

As faculty and students became more accustomed to technology-infused classrooms, we noted increased appreciation for the benefits. One faculty member, in particular, lauded the ‘beauty’ of modularized course software, saying,

The best thing that did come out of [reform]… is the modularized class … It basically has an AI, so artificial intelligence tests the students right in the beginning. And the beautiful feature about it is, as the student goes through it, if it connects a deficiency, it will take them back.

Another faculty member commented on how the software actually helps her address the varying levels of student preparation in her classroom, likely present because of SB 1720:

I have such an extreme range in that class between my students who are behind the 8 ball from day one, to ones that just need a quick refresher and can move on … That’s why I like ALEKS for that level: because it accommodates those differences. And students can really focus on what they need to work on, versus having to do what everybody else does at the same pace.

In much the same way, some faculty members also pointed out that computer software helped them support ELL by presenting course materials in Spanish and Creole. Such features seem simultaneously to have increased social efficiency and democratic equality in unexpected ways.

This appreciation was not universal, even 4 years after implementation. An advisor at one college blamed ongoing student struggles with SB 1720, in part, on a lack of computer literacy skills:

Adult students … they’re completely lost … We have … students with different learning styles and challenges … and we forget that we are not a university. We don’t have those students who are so prepared.

In clarifying her perspective, this participant pointed to McGraw Hill’s ALEKS software, a computer-based assessment and learning system that lends structure to many of the state’s modularized courses: ‘They [adult students] do not like ALEKS, you know?’ What students like and do not like is not always the concern of campus personnel but, in this case, such feelings are relevant to our examination of SB 1720 and its emphasis on technology.

## Discussion

The central questions presented in this article ask how campus personnel articulate the mission of community colleges and how they perceive the mandates of SB 1720 to have shaped their ability to carry out that mission. In answering the first question, we found that FCS personnel affirmed the democratic mission of community colleges previously highlighted by education scholars (Brint & Karabel, 1989; Dowd, 2003; Labaree, 1997). In the words of our participants, equality is embodied in the open access nature of their respective institutions. As noted by one faculty member, this mission means that ‘wherever you are on the continuum, we will take you where you are, and we will help you.’ We also found that campus personnel largely felt the passage of SB 1720, which prioritized social efficiency, made their pursuit of democratic equality more challenging.

When articulating their concerns with social efficiency, participants pointed to acceleration and technology. Past research has shown that unequal access to technology and the skills to use it can be a barrier for low-income and academically underprepared students in their pursuit of postsecondary education (Bell & Federman, 2013). Our findings lend support to these conclusions and highlight additional groups of affected students: those at rural-serving colleges and returning adults. Acceleration, on the contrary, is widely regarded as a best practice in DE reform (Bickerstaff et al., 2016; Hern, 2010; Jaggars et al., 2015). Although this may be true, our participants identified certain groups of students who may not benefit from acceleration, including ELL, individuals with disabilities, student parents, and those who are working.

The passage of time has played some role in changing the perceptions of campus personnel regarding the impact of SB 1720 on college mission. Between 2014 and 2018, some of our participants grew more comfortable with technology and acceleration, and now see how these features of reform might benefit students and complement a mission of both equality *and* efficiency, at least for some student groups. The comments of others reflected ongoing concern. Even still, campus personnel reported that they have maintained their personal commitment to democratic equality through hard work and dedication.

These findings contribute to educational scholarship in several important ways. For one, they provide a contemporary example of the tension that exists between the 2, competing public goals of education—democratic equality and social efficiency— written about by Labaree (1997), and they suggest that, despite initial concern, SB 1720 may have found a way to advance, or at least maintain, both goals. Also, these data give voice to the perceptions of campus personnel, an important group of stake-holders who were responsible for implementation of DE reform, and ultimately, how success or failure played out. The perceptions of implementers have previously been researched, primarily as they pertain to other reforms like No Child Left Behind (e.g., Sunderman, Tracey, Kim, & Orfield, 2004). Our work sheds light on individuals working within a new context of higher education and DE reform. Moreover, our findings reveal that teaching and advising on community college campuses is deeply motivated by personal values and a commitment to underserved students. The campus personnel in our sample spoke at length about finding identity and purpose in the work that they do: expanding educational opportunities to all students, regardless of their incoming level of academic preparation. Finally, these findings call into question social policy that universally promotes accelerated and computer-based courses without considering that some students may require more time to complete coursework or additional supports around technology.

It is important to note the challenge of disentangling the impact of SB 1720 from the local influence of individual colleges. Although the legislation provided a framework for implementation, a good deal of discretion was given to FCS institutions when it came to enacting specific provisions. The experiences of campus personnel reflected here were certainly shaped by the culture of their respective institutions and leadership, as well as the legislation. That being said, the sentiments we include reflect consensus across more than one participant type and institution, reaffirming the validity of our findings.

### Recommendations

Prior to SB 1720, there were significant concerns about DE and the number of students who dropped out (H. M. Levin & Calcagno, 2008; Smith, 2015). Although reform of DE was necessary, many participants shared that certain student populations need more time to complete remediation than newly implemented, accelerated courses afforded. A number of other curricular reforms are being piloted across the nation with preliminary success that may provide an alternate solution for those who are not comfortable with completing a semester of remedial math in 6 or 8 weeks. Such reform efforts include rethinking traditional math pathways that require Algebra (e.g., Hoang, Huang, Sulcer, & Yesilyurt, 2017) and moving toward offering more corequisite coursework (e.g., Belfield, Jenkins, & Lahr, 2016).

Technology will continue to play a major role in how community colleges function during the 21st century (J. S. Levin, 2000). It is not the responsibility of FCS institutions to teach students how to use their computer or email. Yet, we contend it is critical that these colleges recognize the presence of a digital divide and address it as much as possible if they are to continue in their pursuits to facilitate democratic equality, especially since SB 1720 put technology at the center of DE reform. Community colleges must find a way to assist low-income, nontraditional, and rural students in gaining access to, and confidence with, computers moving forward. This could be accomplished in a variety of ways, including (a) letting students check out laptops and other electronic equipment for short periods of time, (b) providing free computer skills classes, and (c) extending lab hours to evenings and weekends to meet the needs of working students. Although some of the colleges we visited do provide these services, more institutions do not. To decrease the impact of the digital divide, as well as contribute to the education of low income, nontraditional, and rural students, it is important that these resources are universally available.

Moving forward, there are many additional opportunities for future research. For instance, enough time has passed since the implementation of SB 1720 that we can now conduct extensive quantitative analyses to determine whether postsecondary access and success has decreased under SB 1720 for certain groups of underserved students, such as ELL, individuals with disabilities, student parents, and those at rural-serving institutions. Once these outcomes have been established, we can compare them to the perceptions held by campus personnel to identify consistencies and discrepancies. Continued qualitative analyses can also explore how this bill may have affected the morale and work satisfaction of campus personnel, particularly across different professional roles and types of FCS institutions.

## Notes

1 The Florida College System (FCS) comprises 2 community colleges and 26 state colleges. Up until 2012, the state colleges were also designated as community colleges. Because this change is recent, all 28 of the institutions, regardless of their formal name, still operate very much like community colleges. As such, it is reasonable to apply literature and theoretical frameworks previously published about community colleges to our research about developmental education at FCS institutions. The FCS stands in contrast to the State University System (SUS), which comprises 12, 4-year universities.

2 These data are one piece of a multiyear, mixed-methods study of SB 1720. The larger project includes qualitative data gathered from individuals at 21 of the 28 FCS institutions, quantitative data from annual administrator surveys, and student record data Florida’s Education Data Warehouse.

3 Although the sample informing this article is limited to the perspectives of campus personnel employed by the 10 original colleges we visited in 2014-2015, campus personnel at other colleges have since expressed similar sentiments.

## Author Biographies

**Amanda N. Nix** is a Research Faculty member with the Center for Postsecondary Success at Florida State University. Her current research focuses on two curricular reforms at work in the Florida College System—Senate Bill 1720 and math pathways—and the consequences of each, particularly for advisors, faculty, and certain student groups.

**Tamara Bertrand Jones** currently serves as an Associate Professor of Higher Education in the Department of Educational Leadership and Policy Studies at Florida State University. Her research examines the sociocultural influences on socialization during graduate education and the professional experiences of underrepresented populations, particularly Black women, in academia.

**Rebecca L. Brower’s** research focuses on qualitative research methods and institutional policies in higher education, particularly those which facilitate student encounters with difference and foster success for students from underrepresented groups.

**Shouping Hu** is the Louis W. and Elizabeth N. Bender Endowed Professor of Higher Education and the founding director of the Center for Postsecondary Success (CPS) at Florida State University. Hu’s research interests examine issues related to postsecondary readiness, outcomes, and success.
